# AMPK in charge: aerobic glycolysis programs unilocularity in adipocytes

**DOI:** 10.1016/j.jlr.2026.101052

**Published:** 2026-05-05

**Authors:** D.J. Kast

**Affiliations:** Department of Cell Biology and Physiology, Washington University School of Medicine, St. Louis, MO, USA

Metabolic complications of obesity depend not merely on the quantity of adipose tissue but also on the capacity of subcutaneous white adipocytes to safely harbor excess lipid and avert ectopic lipid deposition into secondary storage tissues, like the liver, heart, and pancreas (1). Large-scale human genetics supports the concept that limited adipose expandability/storage-capacity contributes to insulin resistance and dyslipidemia (2). Much of our understanding of adipose tissue function during the pathogenesis of obesity comes from studies of the differentiation of preadipocyte cell lines cultured in vitro. However, these approaches have struggled to achieve the most defining feature of healthy white adipocytes: a single large (unilocular) lipid droplet (LD). In a recent publication in the *Journal of Lipid Research*, Maestri and colleagues identify a specific metabolic program that promotes unilocularity in cultured adipocytes, paving the way for more robust in vitro modeling of white adipose tissue physiology (3).

Unilocularity is an important feature of healthy white adipocytes in vivo*;* most of the LD-associated enzymatic activity occurs at the LD monolayer interface, thus LD size and number (i.e., LD surface area to volume ratio) influence lipid storage capacity, lipase accessibility, coat composition, and contact-site valency. Consequently, large LDs are more effective at shielding triglycerides from lipolysis. LD size is known to be regulated by a network of proteins on the LD surface; structural proteins like the perilipin family (PLIN1-5) and double FYVE domain-containing protein 1 (DFCP1), lipolytic enzymes, like adipose triglyceride lipase, and lipid transferases like cell death–inducing DFFA-like effectors (CIDE A-E) (4). Importantly, these protein networks are regulated by the energy sensing AMP-activated protein kinase (AMPK) and protein kinase A, which contribute to the dynamic coordination of LD size and number (5, 6).

To date, the mechanisms by which LD proteins contribute to unilocularity have been studied mostly through differentiation of fibroblasts (such as mouse 3T3-L1 cells) or adipose stem cells using two-dimensional (2D) culture systems (Fig. 1A). While these cells bear some resemblance to white adipocytes, they remain multilocular and lack the cell–cell contacts found in in vivo adipocytes. Beyond morphology, 2D differentiated adipocytes show several programs not found in vivo, such as increased expression of inflammatory genes and of extracellular matrix components as well as dependence on mitochondrial oxidative metabolism of glucose (7). Several studies improved on this cell culture system through modulating the expression of factors known to promote LD growth of in vivo adipocytes, such as overexpressing CIDEC and/or PLIN1 (8), still, 2D differentiated adipocytes remain transcriptionally distant from the in vivo white adipocytes (3). This led to the conclusion that regulation of the LD machinery alone cannot explain why human adipocyte cultures remain stubbornly multilocular and metabolically atypical.

**Fig. 1:**
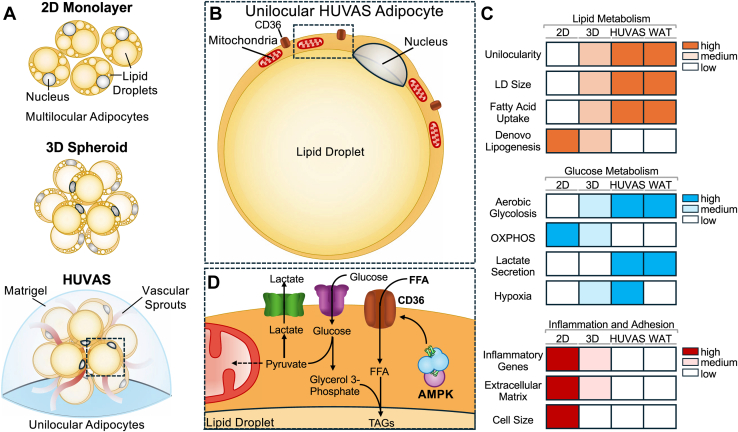
Comparison of culturing systems for adipocyte differentiation. (A) Unlike cultures of 2D monolayers and 3D spheroids, human unilocular vascularized adipocyte spheroid (HUVAS) cultures involve differentiating adipocyte progenitor cells alongside vascular sprouts in a Matrigel scaffold. (B, C) HUVAS adipocytes are more unilocular and have metabolic and transcriptional characteristics that are distant from 2D cultured adipocytes and more closely resemble healthy white adipose tissue (WAT). (D) AMPK in HUVAS plays a central role in regulating fatty acid (FA) flux by mobilizing and activating the lipid transporter Cluster of Differentiation (CD36).

More recently, culturing pre-adipocytes as 3D spherical aggregates (3D spheroids) has been adopted as an effective alternative system that more accurately reflects key morphological and transcriptional features of primary adipocytes (9). However, not all 3D models are equivalent, with substantial differences reported in adipocyte morphology, expression, metabolism, and fatty acid (FA) handling. For instance, it was recently shown that culturing human pre-adipocytes in a 3D environment that mimics the human vascular growth niche during adipogenesis (Fig. 1A) vastly improves differentiation into unilocular adipocytes (Fig. 1B) (10). This raised important questions related to what cellular conditions distinguish unilocular human adipocytes differentiated in a supportive 3D microenvironment from their multilocular counterparts, and how these might be manipulated to control FA uptake and LD morphology.

To address these questions, Maestri and colleagues transcriptionally compared the same population of human subcutaneous stromal vascular fraction cells differentiated by culturing them either as a 2D monolayer, a 3D spheroid, or a 3D spheroid embedded in an extracellular matrix (Matrigel) scaffold. The Matrigel drives stromal vascular fraction endothelial cells to form vascular sprouts and establishes a niche that promotes the differentiation of human unilocular vascularized adipocyte spheroids (HUVAS). They found that HUVAS bear a transcriptional signature that is similar to healthy adipose tissue, whereas 3D spheroids lie between adipocytes and preadipocytes, and 2D adipocytes remain most similar to preadipocytes (3) (Fig. 1C). Maestri and colleagues found that the key difference is centered around two transcriptional programs: glucose metabolism and hypoxia.

The elevated glucose metabolism in HUVAS was surprising, as it was expected that canonical adipocyte and LD markers would correlate with LD size and unilocularity in vitro. Instead, it was found that lactate secretion scales with LD size. This is a compelling observation since primary white adipocytes are well known to convert a substantial amount of glucose into lactate, which correlates with both their size and triglyceride content. Supporting this observation, inhibition during HUVAS differentiation of lactate dehydrogenase A, which converts the pyruvate from aerobic glycolysis into lactate, reduced LD size and unilocularity. Conversely, impairing OXPHOS during differentiation to divert pyruvate into lactate did little to change LD morphology in HUVAS, but markedly improved LD size and unilocularity in 3D spheroids. This is a sharp departure from 2D cultures, where driving aerobic glycolysis does not promote unilocularity and OXPHOS inhibition reduces both LD size and unilocularity.

In addition to elevated lactate secretion, HUVAS also show increased FA uptake capacity in response to AMPK activation (Fig. 1D). Pharmacological activation of AMPK during late maturation increased LD size and FA uptake, whereas AMPK inhibition did the opposite and ultimately impaired maturation in 3D spheroids. Building on this observation, pharmacological inhibition of the FA transporter, Cluster of Differentiation 36, reduced droplet size/unilocularity, indicating that HUVAS, unlike 2D adipocytes, depend on exogenous FA uptake rather than de novo lipogenesis as the dominant route for building large LDs. Perhaps, the most diagnostic experiment is that AMPK activation could restore unilocularity and FA uptake, even when lactate-producing glycolysis is inhibited, positioning AMPK downstream of aerobic glycolysis and upstream of FA uptake. While the role for AMPK in HUVAS is clear, it differs from white adipose tissue, where activating AMPK can impair insulin-stimulated lipogenesis, while impeding lipolysis through AMPK-dependent phosphorylation of hormone-sensitve lipase (11). These seemingly opposing roles of AMPK highlight the complex metabolic relationship of AMPK and lipid storage. Further investigation into the specific cellular conditions that lead to AMPK activation, such as changes in AMP/ATP ratio, NADH/NAD^+^ redox state, and in upstream kinases, will be needed to pin down the mechanism involved and to determine the relevant pathway for white adipocytes in vivo.

Maestri and colleagues found that several hypoxia-related genes are significantly elevated in HUVAS (Fig. 1C), suggesting a potential role for hypoxia-inducible factor 1 alpha (HIF-1α) in generating “healthy” unilocularity. Highlighting a potential role for hypoxia in adipocyte differentiation is timely as human obesity has been linked to altered adipose oxygenation and elevated HIF-1α signaling—a key driver of adipose dysfunction (12). Recent modeling and systems-level work support the idea that HIF-1α can exert temporally separate and opposing roles in adipocyte lipid accumulation, depending on the differentiation state and context. For example, hypoxia downregulates key genes of preadipocyte differentiation in 2D cultures while simultaneously promoting lipid accumulation by impairing the LD-associated lipolytic machinery (13). However, hypoxia gene expression in HUVAS was surprising as vascular sprouts provide greater oxygen access. Indeed, prolonged HIF-1α activation did not improve unilocularity and instead reduced LD size. Consequently, the role of hypoxia in HUVAS remains elusive and may be a case of mistaken identity, as hypoxia shares several transcriptional signatures with aerobic glycolysis.

A defining feature of HUVAS is the presence of the Matrigel scaffold. The Matrigel promotes expansion of the adipocytes found in the spheroid and these enlarged adipocytes have large LDs, suggesting that LD size was correlated with adipocyte size (10). However, it is worth noting that the Matrigel imposes mechanical stiffness on the spheroid that may facilitate transcriptional changes that influence cell signaling and metabolism. It is well appreciated that more ECM stiffness drives nuclear localization of Yes-associated protein transcriptional co-activator with PDZ-binding motif and promotes aerobic glycolysis in cancer cells and in diseased cardiomyocytes (14). Interestingly, Yes-associated protein is inhibited by AMPK-dependent phosphorylation. Thus further work is needed to unravel the interdependence of mechanical stiffness and aerobic glycolysis in white adipocyte function.

The molecular insights offered by Maestri *et al.* highlight the importance of the interplay between culture conditions, metabolism, and adipocyte LD dynamics. The work also implies that “unilocularity” is not merely a cosmetic marker for differentiation but rather reflects a finely tuned metabolic program. Naturally, this carries practical consequences for interpreting decades of adipocyte work conducted in multilocular 2D systems. For example, if 2D adipocytes are biased toward inflammatory/fibrotic transcriptional activation and distinct substrate routing, then mechanistic conclusions about lipid handling may depend on the microenvironment and not the adipocyte itself. While no in vitro culture system can truly replicate the ever evolving metabolic and transcriptional landscapes that prescribe white adipocytes, this work offers a powerful new approach for establishing an in vitro culture model that more faithfully recapitulates white adipocyte cell biology and will no doubt have tremendous value in studying the mechanisms and pathways important for adipocyte metabolism in health and disease.

## Conflicts of interest

The author declares that they have no conflicts of interest with the contents of this article.
